# The effect of the menstrual cycle on dichotic listening

**DOI:** 10.1371/journal.pone.0212673

**Published:** 2019-02-22

**Authors:** Richard J. Morris, Erin M. Ingvalson, Michael P. Kaschak, Alissa N. Smith

**Affiliations:** 1 School of Communication Science and Disorders, Florida State University, Tallahassee, Florida, United States of America; 2 Department of Psychology, Florida State University, Tallahassee, Florida, United States of America; University College London, UNITED KINGDOM

## Abstract

The purpose of this study was to determine the effect of the menstrual cycle on responses to a dichotic listening task. It was hypothesized that participants would exhibit a stronger right ear advantage during the menstrual cycle days when estrogen levels are at their peak. It was also hypothesized that the women not taking oral contraceptives would exhibit greater variations in ear advantage over the course of their menstrual cycle than those taking oral contraceptives. Finally, it was hypothesized that the error response rates would remain similar across different listening conditions and over the menstrual cycle. The participants were 30 women who took oral contraceptives and 15 who did not. They completed nine listening sessions comprised of three dichotic listening tasks: forced-left, forced-right, and open. The data were analyzed using a mixed effects models. The participants exhibited a reduction in right ear responses on the days that corresponded to when the level of estrogen would begin to increase. This response was different from what had been hypothesized. The analysis also indicated no response differences between the two groups of women. In addition, the women exhibited fewer errors over the course of the sessions, implying that they adapted to the task. The results indicate that the women’s hormone fluctuation across the menstrual cycle affected their responses to the forced-left, cognitive control, task only.

## Introduction

Results of dichotic listening studies consistently indicate lateralization of speech perception [1, 2]. However, opinions differ as to the operations underlying these results with some researchers preferring an attention theory approach and others preferring a structuralist-localizationist one [3, 4, 5]. Regardless of the underlying operations, dichotic listening activities provide a non-invasive means for investigators to evaluate language lateralization [1]. Dichotic listening activities involve the simultaneous presentation via headphones of different syllables to the left and right ears and prompting a listener to indicate the syllable heard most clearly [6]. Listeners completing dichotic exercises generally report hearing the syllable presented to the right ear, or a right ear advantage (REA). Two dichotic listening based explanations have been proposed for REA. The first relates to the human ability to selectively attend to stimuli from each ear separately that has demonstrated a generally occurring verbal task based attentional bias toward the right side [1, 7, 8]. The other relates to structural lateralization of language in the left hemisphere of the brain [2, 5, 9].

Researchers have developed different conditions during dichotic listening tasks to assess their effects on participant responses. For example, some researchers introduced additional listener attention or cognitive control to the dichotic listening task by directing the listeners’ attention to the syllables presented to a specific ear. Attention is directed to the syllables presented to the left ear, a forced left ear response, in order to analyze cognitive control processes; conversely, directing the listener to attend to the syllables presented to the right ear, a forced right ear response, is used to analyze selective attention [1, 9, 10]. The cognitive control in the forced left ear condition has been stated as the participant providing an instruction-based rather than a stimulus-based response. In addition, researchers have used dichotic listening tasks to assess variations in the strength of REA across the hormonal changes that occur in the menstrual cycle [9, 11,12, 13, 14, 15].

The fluctuation of hormonal levels over the menstrual cycle causes changes in many different systems of the body. Previous studies suggest that variations in ovarian hormones during the menstrual cycle may produce positive effects on a number of cognitive functions including tasks involving working memory, cognitive inhibition, and verbal memory [9]. The ovarian hormone variations also have been shown to affect the function of the auditory mechanism [16, 17, 18]. Together, estrogen and progesterone may directly affect steroid receptors in the cochlea and indirectly influence auditory function by modulating cochlear blood supply [17]. For example, Batta et al. [18] reported significantly shorter latencies of auditory brainstem evoked responses when their participants were tested during the days of their menstrual cycles after ovulation. In contrast, Hu and Lau [14] reported longer latency times when their participants were tested during this same portion of the menstrual cycle.

Researchers have reported contrasting results for the effects of the ovarian hormone changes across the menstrual cycle on results from dichotic listening studies. Some have reported that higher levels of the ovarian hormones estrogen and progesterone are associated with increases in REA [12, 15]. These researchers found a shift in REA among women across phases of the menstrual cycle that affected their responses to the dichotic stimuli. The women in their studies exhibited lower REA scores during the days of menstrual flow and over the following week when ovarian hormones were at their lowest levels compared to scores collected during the days just preceding and just following ovulation. However, other researchers found that their participants exhibited no hormonally based changes across the menstrual cycle to open and forced right ear response dichotic listening tasks, but reductions in REA for a forced left ear response condition during the week before ovulation [9, 14]. The authors of these studies concluded that increased levels of ovarian hormones increased cognitive control, but did not affect selective attention or an open response condition. Finally, response differences occurred between different phases of the menstrual cycle when more linguistically complex stimuli were used, such as staggered spondaic words and CVC rhyming words [11, 19]

Hu and Lau [14] recorded auditory brainstem responses at four times in the menstrual cycle and for the three dichotic listening conditions, open forced left ear response, and forced right ear response. They found a lower REA in dichotic listening responses for the forced left ear condition measured during the pre-ovulation portion of the menstrual cycle. This increase in lateralization toward the left side coincided with longer brainstem response latencies for peak V of the auditory signals [14]. In contrast, other researchers reported that higher levels of estradiol were associated with lower lateralization scores across all dichotic listening conditions [11, 13]. In one of these studies Hodgetts et al. [13] used a different method for calculating ear advantage. Rather than focusing on the right ear, these authors calculated the absolute value of lateralization and thus the percentage of left ear advantage that occurred in the forced left ear response condition was combined with any REA that occurred under any condition [13].

When calculating REA, the authors of the above studies have focused on the extent to which the listeners correctly identified the target, noting only whether that target was presented to the left or to the right ear. However, there are trials in which listeners indicate they hear a third sound, presented to neither ear, which is best coded as an error. If researchers were to include these error responses in analyses, it is possible these measures could provide additional insight concerning the effects of ovarian hormone levels on REA. In particular, it may be that there would be more errors in a forced-left condition than in a forced-right or in an open condition, demonstrating more difficulty attending to the left ear as the correct left ear response requires greater cognitive control. If an increase in ovarian hormones is associated with an increase in REA, an increase in error rates in a forced-left condition might occur when these hormones are at their peak level.

Researchers of the interaction between dichotic listening results and the phase of the menstrual cycle and associated ovarian hormone levels have used differing methods to determine their participants’ menstrual cycle phase. Some researchers had their participants report the day of the most recent onset of menstruation as the marker of the beginning of her cycle and then counted the days [e.g., 12], other researches collected hormone assays from saliva [e.g., 13], and others have used basal body temperature [14]. When Hodgetts et al. [13] reviewed several studies, they found similar dichotic listening results across methods for determining menstrual phase. From that report, Hu and Lau [14] concluded that any of the previously reported methods can be acceptable for investigating the interaction between ovarian hormone levels and dichotic listening results.

In addition, oral contraceptives (OC) may affect women’s responses to dichotic listening tasks by stabilizing the levels of estrogen and progesterone across the menstrual cycle. Thus, these women’s responses can serve as a control group to which any hormone-related changes in dichotic listening could be compared. Currently, there are no data illustrating how OCs might affect responses to dichotic listening tasks. The shifts in the estrogen and progesterone levels from OCs may relate to changes in dichotic listening responses.

The objective of the current study was to explore more thoroughly the previous reports of the effect of the phases of the menstrual cycle on dichotic listening responses. First, it seemed reasonable to extend the length of the study to include two menstrual cycles rather than the single cycle included in the previous studies. This extension would provide data that could indicate the stability and reliability of the women’s responses. This method contrasts with that of Hodgetts et al. [13] who controlled for practice effects by using a between-subjects design and had their participants complete the dichotic listening tasks one time. Although that design provided information on the effects of different hormone levels between women, it could not provide information on the effects of the hormone changes through the menstrual cycle. For this study it was hypothesized that the participants in the study would exhibit a stronger REA during the days before ovulation when estrogen levels peak. Second, further evidence was sought concerning the effects of the menstrual cycle on the dichotic listening responses by examining performance in women taking oral contraceptives (OC) and women not taking oral contraceptives (NOC). Since taking OCs can stabilize the ovarian hormones across the menstrual cycle, it was hypothesized that the NOC using women would exhibit greater variations in ear advantage over the course of their menstrual cycle than the OC using women. This finding would reinforce the reports from previous studies of an interaction between ovarian hormone levels and REA. Finally, in order to utilize all of the data, error responses would be included in the measurements across the experimental conditions and across the sessions. It was assumed that error responses would be relatively consistent across the experimental conditions and the sessions.

## Methods

### Participants

Participants included 53 healthy young females who were recruited from a communication disorders undergraduate course. The participants were in two groups, 35 who took oral contraceptives (OC) and 18 who did not (NOC). Each participant had passed a screening test within the past three months that indicating that she had normal hearing. The participants were offered extra credit in a course for their participation. To encourage repeated participation, the extra credit points were awarded separately for each session throughout the research process. This project was approved by the Institutional Review Board at Florida State University on 23 September 2014 (HSC # 2014.13183).

Participants were removed from the sample if they missed three or more listening sessions. This criterion resulted in a loss of five participants from the OC group with 30 remaining and a loss of three participants from the NOC group with 15 remaining.

Durations of the women’s menstrual cycles were determined by participant self-reporting at the beginning of each experimental session. Of the 30 OC women, 28 used versions with a 28-day cycle, although some of these women reported being at days 29 to 33 of their menstrual cycle. These women used a variety of forms of OC, 12 used monophasic, 6 used biphasic, and 11 used triphasic. Two women used extended cycle contraceptives, one took a monophasic OC with a 91-day menstrual cycle and the other had a monophasic hormonal implant. The woman using the extended cycle OC began a cycle between the first and second experimental session. The woman with the hormonal implant began the study at day 89 of her cycle and ended at day 144. Of the 15 NOC women, 14 had menstrual cycle durations in the normal range, 26–33 days. One woman had a menstrual cycle of 43 days.

### Procedures

Participants completed nine listening sessions of approximately 15 minutes each, with one session per week. The nine listening sessions over nine weeks were used in an attempt to record data during two complete menstrual cycles. The listening sessions were conducted on the same day of the week and same time of day in a computer classroom. The initial session was approximately 30 minutes so that the participants could complete paperwork and be trained on the experimental task. During the initial session, the participants completed the informed consent form and a brief history form that included information on their use of oral contraceptives. At the beginning of every following session the participants completed a brief form that included having them write the date of the onset of their last menstrual cycle. The length of an individual participant's menstrual cycle was counted starting with the first day of menstrual bleeding.

The dichotic listening tasks were completed using the Alvin software package for speech perception research [20]. The stimuli were sound file recordings of an adult male saying the syllables /bɑ/, /dɑ/, /gɑ/, /pɑ/, /tɑ/, and /kɑ/ in a General American Accent with constant amplitude and frequency and 400–450 ms durations. For each presentation the Alvin software directed two of the syllables to the participants, one in each ear. Each experimental condition included 66 randomly selected pairs of stimuli, 60 with different stimuli for the two ears and 6 with the same stimuli for both ears. The same stimuli presentations were included to assess whether the participants were attending to the task. The same stimuli presentations were not included in the measurements of the participants’ responses.

The stimuli were presented via Sennheiser HD-201 stereo headphones. Each participant set the signal level at her own comfortable loudness level [21]. For every presentation, the participant selected the plosive she heard from a choice of the six possible CV pairs by pressing the appropriate key on the computer keyboard. Once the plosive was selected, the next stimulus was presented. The participants were instructed to be thoughtful and deliberate in their selections and were given the choice to listen to the individual stimulus more than once.

The three experimental conditions of the Bergen dichotic listening test [2] were used: open response, meaning that the participants indicated the CV that was most clearly heard; forced left ear response, meaning that the participants were to attend to and indicate the signal presented to the left ear; and forced right ear response, meaning that the participants were to attend to and indicate the signal presented to the right ear. Each condition contained 66 presentations of the CV stimuli. At every session, the participants completed all three conditions. To limit practice effects, the order of the conditions was varied systematically every week. It took approximately three minutes for each participant to complete one set of stimuli. At the completion of each set the experimenter would wait until all participants were finished before indicating the next listening condition.

The percentage of correct left ear responses, correct right ear responses, and error responses were calculated. In order to compare the results of the present study with previous dichotic listening research laterality quotients were calculated for the participants’ responses using the equation [(RE—LE) / (RE + LE)] x 100 [e.g., 13].

### Analysis

Three mixed effects analysis models were developed to determine the effects of probability of conception, oral contraceptive use, and task on response rates for left ear, right ear, and error rates. The response data were coded based on whether the participants had chosen the phoneme that matched the stimulus presented to the left ear, the stimulus presented to the right ear, or an error, a plosive that did not match one of the two correct response options. The data were entered into the models, one for each type of response. The mixed effects models were constructed using the lme4 package [22] implemented in R [23]. In all models, the fixed factors were session, contraceptive group (oral contraceptive user or non-oral contraceptive user with the reference level set to the non-oral contraceptive user), condition (forced left, forced right, or open with the reference level set to the open condition), the interaction of session and condition, and probability of conception. Subject was included as a random intercept and as a random slope within session and condition and within the session by condition interaction. The Kenward-Roger approximation was used to generate *p*-values.

In addition, the probability of conception by day of the menstrual cycle was calculated using the method described by Stirnemann, et al., [24]. These authors used a Pearson product-moment correlation to associate the day of the menstrual cycle with percentages of conception from a sample of 5830 women. They provided a table with the probability of conception for every day of the menstrual cycle from day 1, the first day of menstruation in a menstrual cycle, to day 35. As the probability of conception was 0% for days 31–35 [24], menstrual cycle days beyond 35 were assigned a probability of 0%. This range allowed for a variety of normal periodic menstrual cycle durations. Using the probability of conception was preferable to using phases of the menstrual cycle as there are some that have two phases, follicular and luteal, some that have three phases, and some that have four phases. The differing number of menstrual cycle phases result in certain days of the menstrual cycle occurring during different phases in different studies. Using probability of conception avoided that issue. The days of the menstrual cycle that Stirnemann and colleagues [24] reported having the highest probability of conception matched Cole, Ladner, and Byrn’s [25] report on the days of the menstrual cycle most likely for ovulation. For the present study, the probability of conception by day of the menstrual cycle was used to represent the relative level of ovarian hormones for each day of the cycle.

The reliability of the listeners’ responses was determined using the matched syllable presentations. The listeners exhibited good reliability with 78% correct responses on the reliability stimuli for the forced left ear and forced right ear conditions and 76% correct responses on the reliability stimuli for the open response condition.

The data for the two participant groups across the three presentation conditions and their laterality are available in S1 File.

## Results

The percentage means of right ear syllable responses, left ear syllable responses, and incorrect responses were compared across the different OC types, monophasic, biphasic, and triphasic, for the three presentation conditions. As shown in Table 1, the women taking all of the OC types responded similarly in all three conditions. Their mean response ranges were 28.3% to 32.3% for left ear stimuli, 38.6% to 45.4% for right ear stimuli, and 22.9% to 32.0% for error responses. These similarities allowed the combining of the OC women into a single group of OC participants.

**Table 1:** Percent plosives selected for each ear as a function of condition and type of oral contraceptive.

	Forced Left			Forced Right			Open		
Response	Mono	Bi	Tri	Mono	Bi	Tri	Mono	Bi	Tri
Left	32.3	30.5	30.1	31.1	31.1	29.5	31.8	30.5	28.3
	*(11*.*3)*	*(12*.*7)*	*(9*.*9)*	*(11*.*4)*	*(14*.*3)*	*(10*.*3)*	*(11*.*3)*	*(12*.*7)*	*(10*.*8)*
Right	41.7	42.0	40.4	44.8	42.4	38.6	45.4	42.6	41.0
	*(12*.*3)*	*(13*.*9)*	*(15*.*1)*	*(12*.*8)*	*(14*.*3)*	*(10*.*8)*	*(12*.*2)*	*(13*.*6)*	*(11*.*4)*
Error	26.0	27.5	32.0	24.1	26.5	31.9	22.9	26.9	30.6
	*(10*.*6)*	*(9*.*2)*	*(10*.*3)*	*(10*.*2)*	*(9*.*0)*	*(9*.*5)*	*(9*.*6)*	*(9*.*1)*	*(8*.*8)*

Mean values are in Roman and standard deviations are in parentheses and italics. Mono is for those taking monophasic OC, bi is for those taking biphasic OC, and tri is for those taking triphasic OC.

**Fig 1:**
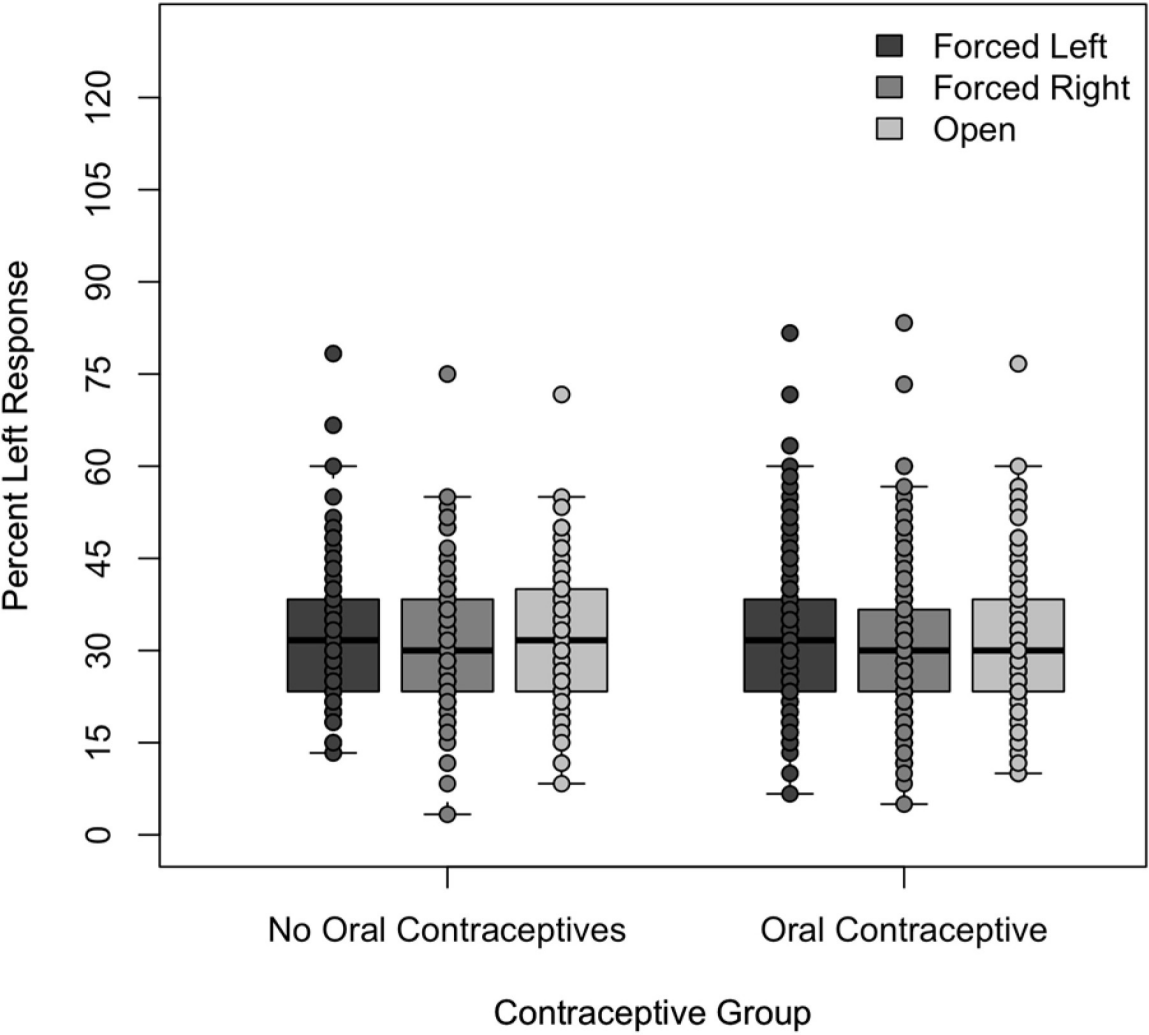
Percentage of left ear responses under the three listening conditions to the two listening groups. Percentage of left ear responses under the forced left, forced right, and open conditions by the women not taking oral contraceptives and those taking oral contraceptives.

**Fig 2:**
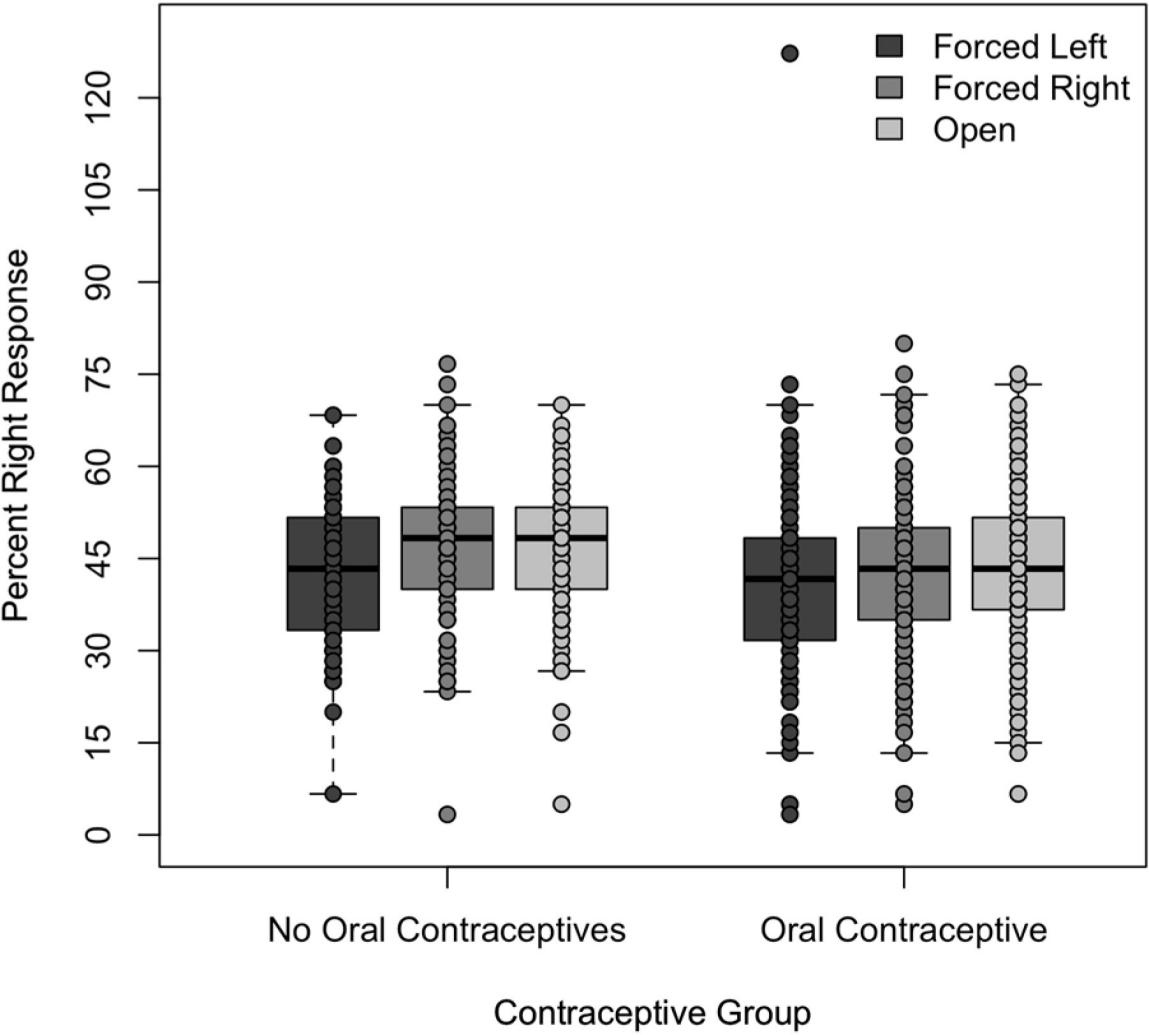
Percentage of right ear responses under the three listening conditions to the two listening groups. Percentage of right ear responses under the forced left, forced right, and open conditions by the women not taking oral contraceptives and those taking oral contraceptives.

**Fig 3:**
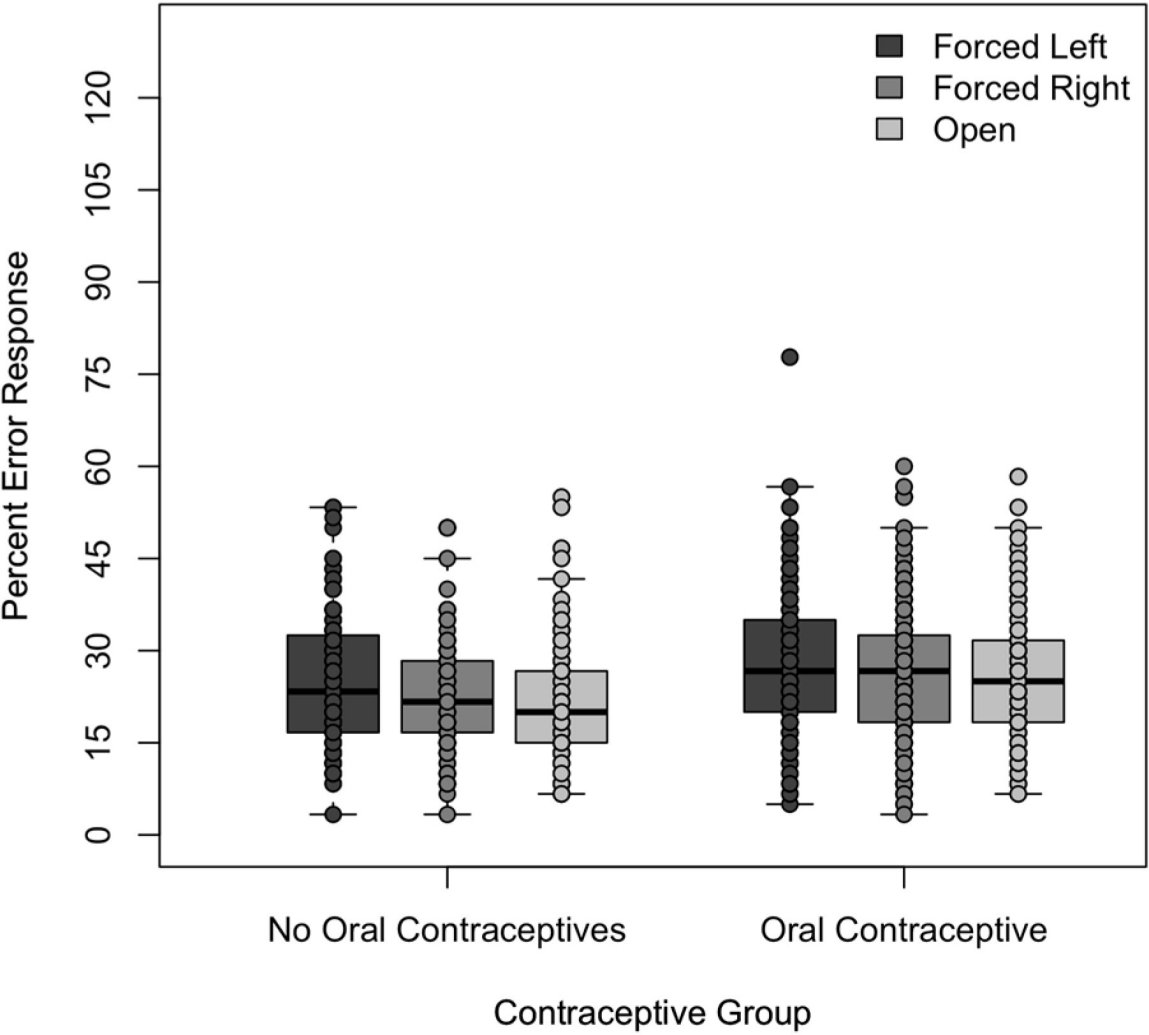
Percentage of error responses under the three listening conditions to the two listening groups. Percentage of error responses under the forced left, forced right, and open conditions by the women not taking oral contraceptives and those taking oral contraceptives.

The percentage means of right ear syllable responses, left ear syllable responses, and incorrect responses were compared for the three presentation conditions. As shown in Figs 1–3, both groups of participants responded similarly under all three conditions. Their selection of the stimuli presented to the left ear ranged from a mean of 30.6% to 31.8% for the OC group and 30.1% to 32.4% for the NOC group. Their selection of the stimuli presented to the right ear ranged from a mean of 41.2% to 43.8% for the OC group and 42.6% to 47.1% for the NOC group. Finally, their selection of error plosives ranged from a mean of 25.5% to 27.6% for the OC group and 21.2% to 25.0% for the NOC group. Mean responses for each ear as a function of condition and oral contraceptive use can be seen in Table 2.

**Table 2:** Percent plosives selected for each ear as a function of condition and oral contraceptive use.

	Forced Left		Forced Right		Open	
Response	OC	NOC	OC	NOC	OC	NOC
Left	31.79	32.37	30.74	30.59	30.64	31.83
	*(11*.*46)*	*(10*.*90)*	*(11*.*57)*	*(10*.*82)*	*(11*.*04)*	*(10*.*77)*
Right	41.19	42.59	42.80	47.11	43.84	46.93
	*(13*.*39)*	*(11*.*07)*	*(12*.*52)*	*(11*.*82)*	*(12*.*02)*	*(10*.*82)*
Error	27.66	25.00	26.45	22.29	25.51	21.19
	*(10*.*48)*	*(10*.*61)*	*(10*.*54)*	*(8*.*91)*	*(9*.*77)*	*(9*.*34)*

Mean values are in Roman and standard deviations are in parentheses and italics.

The OC women exhibited laterality quotients of 16.7 for the open condition, 11.5 for the forced left condition, and 16.2 for the forced right condition. The NOC women exhibited laterality quotients of 19.9 for the open condition, 14.4 for the forced left condition, and 21.4 for the forced right condition. These laterality quotient scores are in the range of those reported in previous studies.

The mixed effects model output for the left ear responses are shown in Table 3. None of the fixed factors were significant predictors of left ear responses.

**Table 3:** Parameter estimates from the mixed-effects model for left ear responses.

	*b*	*SE*	*t*	p*
Intercept	31.09	1.93	16.10	0.00
Testing Session	0.07	0.19	0.37	0.71
Forced Left Condition	-0.27	1.40	-0.19	0.85
Forced Right Condition	-1.21	1.40	-0.86	0.39
Oral Contraceptive User	-0.71	2.02	-0.35	0.72
Probability of Conception	0.00	0.01	0.45	0.66
Testing Session x Forced Left Condition	0.24	0.25	0.96	0.34
Testing Session x Forced Right Condition	0.18	0.25	0.70	0.48

*Note: p-values were obtained via the Kenward-Roger approximation.

**Fig 4:**
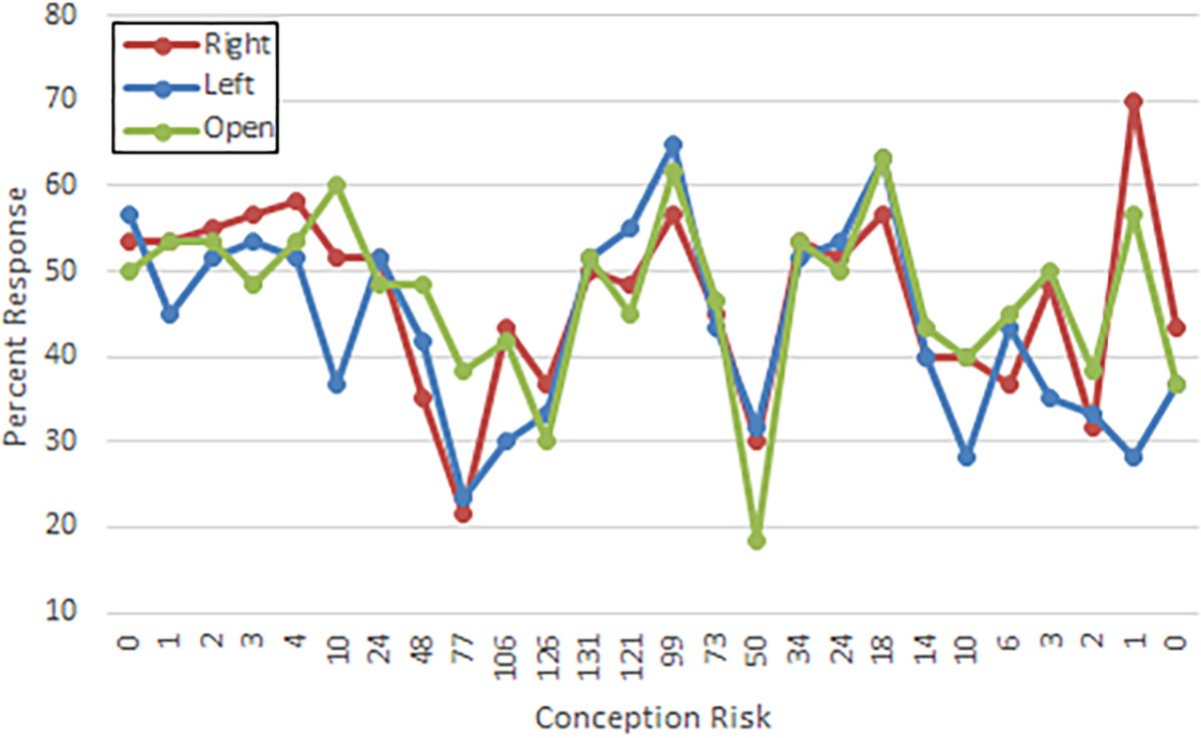
Percentage of right ear responses as a function of risk of conception. Mean percentage of right ear responses at each conception risk level across the month for the forced right ear, forced left ear, and open conditions.

Significant main effects were found in the mixed effects model output for the right ear responses. As can be seen in Table 4, the listeners produced significantly fewer right ear responses in the forced left condition than in the open condition (*b* = -4.28, *SE* = 1.62, *t* = -2.65, *p* = .01). Table 4 also shows that the right ear responses did not significantly differ between the other two conditions, forced right and open. As shown in Fig 4, as the probability of conception increased during the days prior to probable ovulation when estrogen levels rise, the percentage of right ear responses decreased (*b* = -0.01, *SE* = 0.01, *t* = -2.43, *p* = .02). There also was a decrease in the percentage of right ear responses after the days of probable ovulation when estrogen levels rise to a lesser extent.

**Table 4:** Parameter estimates from the mixed-effects model for right ear responses.

	*b*	*SE*	*t*	p*
Intercept	45.17	2.35	19.19	0.00
Testing Session	0.36	0.21	1.70	0.09
Forced Left Condition	-4.28	1.62	-2.65	0.01
Forced Right Condition	-1.02	1.47	-0.69	0.49
Oral Contraceptive User	-2.56	2.36	-1.08	0.28
Probability of Conception	-0.01	0.01	-2.43	0.02
Testing Session x Forced Left Condition	0.23	0.27	0.85	0.40
Testing Session x Forced Right Condition	0.07	0.26	0.27	0.79

* p-values were obtained via the Kenward-Roger approximation.

**Fig 5:**
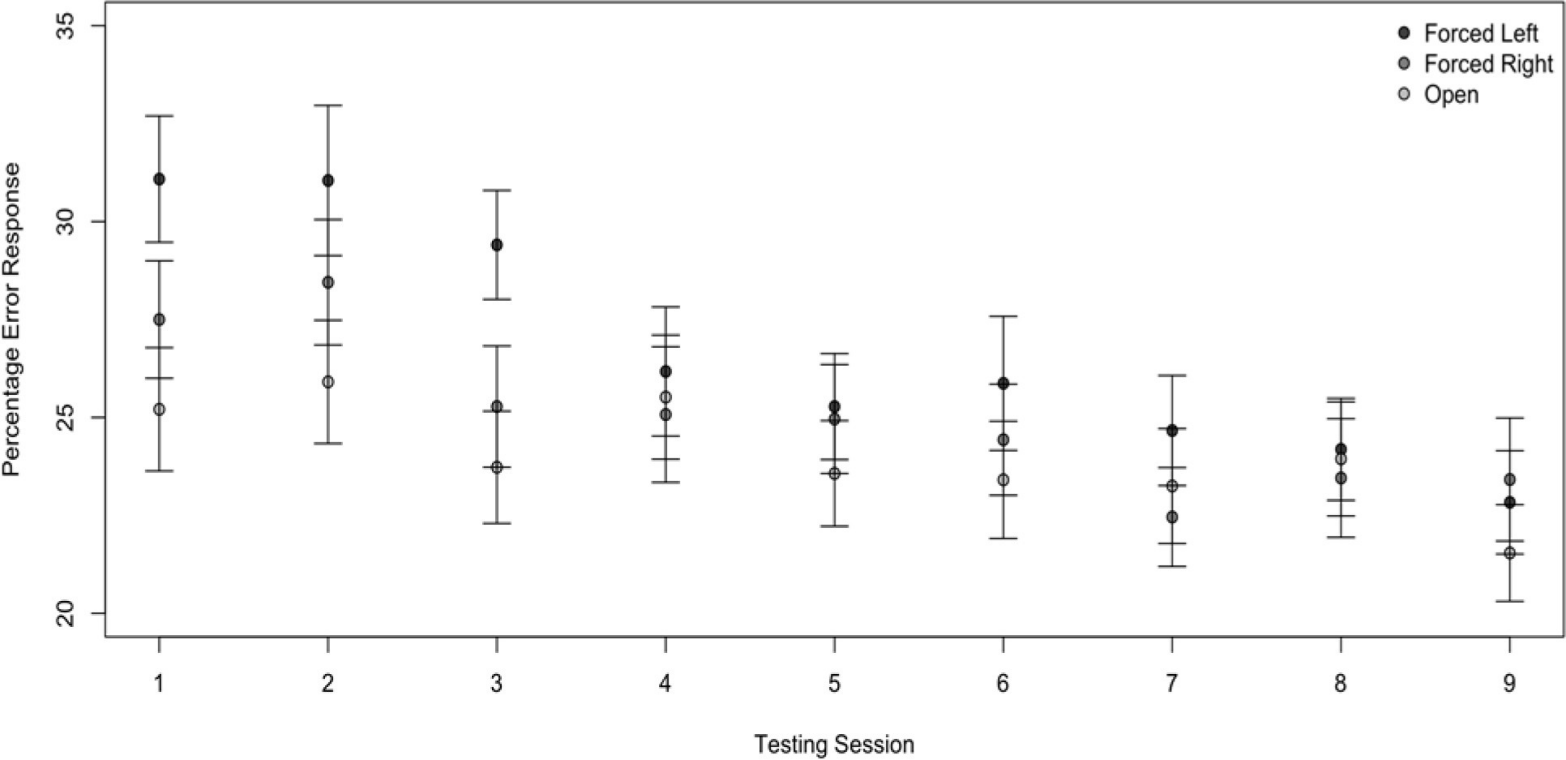
Percentage of error responses as a function of listening condition and testing session. Mean and standard error of the error responses for the three listening conditions over the nine testing sessions.

The mixed effects model output for the error rates are shown in Table 5. This analysis revealed significant main and interaction effects for the error responses. The participants made more errors in the forced left condition than in the open condition (*b* = 5.84, *SE* = 1.27, *t* = 4.61, *p* < .001); however, their error rates did not differ significantly between the forced right and the open condition. There also was a significant main effect with the error rate increasing as the probability of conception increased (*b* = 0.01, *SE* = 0.004, *t* = 2.56, *p* = 0.01). Another significant main effect occurred over the sessions, with the participants making fewer errors in later testing sessions (*b* = -0.43, *SE* = 0.19, *t* = -2.31, *p* = 0.02). Finally, there was an interaction between the sessions and condition (*b =* -.64, *SE* = 0.20, *t* = -3.18, *p* < .001). As shown in Fig 5, the error rate decreased across sessions for the forced left condition more than for the open condition, but the decrease in error rate for the open condition and the forced right condition did not differ.

**Table 5:** Parameter estimates from the mixed-effects model for error responses.

	*b*	*SE*	*t*	p*	
Intercept	24.89	2.20	11.33	0.00	
Testing Session	-0.43	0.19	-2.31	0.02	
Forced Left Condition	5.84	1.27	4.61	0.00	
Forced Right Condition	2.09	1.08	1.93	0.06	
Oral Contraceptive User	1.68	2.28	0.73	0.47	
Probability of Conception	0.01	0.00	2.56	0.01	
Testing Session x Forced Left Condition	-0.64	0.20	-3.18	0.00	
Testing Session x Forced Right Condition	-0.22	0.18	-1.20	0.23	

*Note: p-values were obtained via the Kenward-Roger approximation.

## Discussion

The purpose of this study was to examine the effect of the phases of the menstrual cycle on women's responses to a set of dichotic listening tasks. The dichotic listening tasks in this study were used to measure listener responses under three different conditions: free response, forced left ear response, and forced right ear response. The hypothesis that the participants of the study would exhibit a stronger REA during the days of higher estrogen level before ovulation was not supported. In fact, the results showed that the proportion of right ear responses decreased when estrogen levels began rising during the days prior to ovulation and after ovulation when estrogen levels rise again. In addition, listeners showed an effect of learning as error rates decreased across sessions, particularly for the forced-left condition. Hodgetts et al. [13] indicated this learning effect was a problem of repeated measure designs when evaluating dichotic listening responses. The consistent findings across the forced left, forced right, and open conditions after the first few sessions support the concept that the strength of the REA is based on attention [8]. It appears that the listeners’ responses in the present study may have been more affected by their ability to maintain attention to the specific task over the repeated sessions than by their hormonal levels.

These results support the previous findings that REA decreased during the phase of the menstrual cycle when probability of conception and ovarian hormone levels would be highest [11, 13]. Hodgetts et al. [13] reported this effect for their high estradiol group throughout the menstrual cycle and a menstrual cycle pattern for their low estradiol group. Hausmann and colleagues [26] reported reduced transcallosal interhemispheric inhibition during the days of the menstrual cycle when either estrogen or progesterone were high. They reported that these changes resulted in reduced cortical lateralization. They speculated that these hormones worked to reduce glutamate responses in the callosal fibers [26]. The present data indicated the greatest change on the menstrual cycle days when the estrogen levels would begin to rise. This finding is consistent with the previous report that the derivative of ovarian hormone concentration was more important for modeling behavioral changes than the level of the hormone [27].

In addition, the inclusion of participant error rate in the present study allowed for the observation that not only was the REA lower during the time of highest probability of conception, but the participants’ error rate increased. Thus, the present results conflict with those who reported that higher levels of estrogen and progesterone are associated with increases in the REA [12, 15, 28].

One possible reason for the disparity in results between our data and previous studies is the methodological differences between studies. The present study included more participants than many of the previous studies, although the Hodgetts et al. [13] study had more participants. In addition, the data collection in the present study occurred over two rather than one menstrual cycle. The longer data collection time should have provided a better indication of a stable menstrual cycle based response pattern. In addition, the dichotic listening task in the present study included more stimuli than had been used in previous work in this area. Finally, the method of recording the subjects’ responses in the present study allowed them to directly respond. In contrast, the researchers in the previous studies recorded the participant responses; a data recording method that could have added a potential error source to the data collection.

Another key methodological difference that needs to be highlighted is that no previous study included women who were taking oral contraceptives. This is important because of the stabilizing effects of oral contraceptives on ovarian hormones. Because all oral contraceptives shift ovarian hormones in an effort to keep them stable across the menstrual cycle, it was anticipated that the use of an oral contraceptive would limit any ovarian hormone related shift in REA across the sessions. What was unexpected, however, was that there was no difference in REA between the women taking OC and the NOC women. It was expected that the relatively consistent levels of progesterone and estradiol for three of the four weeks of the cycle among those using monophasic OC should have altered the pattern for these women. That the women using different methods and patterns of hormones for contraception had the same patterns of responses to the dichotic listening tasks as the NOC women indicates that these hormones have minimal effect on these tasks.

Previous evidence indicates that together, estrogen and progesterone may directly affect steroid receptors in the cochlea and may indirectly influence auditory function by modulating cochlear blood supply [17]. High concentrations of ovarian hormones consistent with ovulating are also linked with a change in a number of cognitive functions including tasks involving working memory, cognitive inhibition, and verbal memory [9] and some sensory processes, such as increased visual acuity, better color discrimination, and increased hearing sensitivity when estradiol is highest [17]. However, the current results suggest that the increases in ovarian hormone levels during the menstrual cycle lead to a reduction in the REA immediately after ovulation. This finding suggests that the physiological differences in the auditory mechanism that occur during the menstrual cycle do not limit REA. The possibility remains that other aspects of auditory function could be affected by the varying levels of ovarian hormones across the menstrual cycle.

The finding of a lower REA in the forced left condition is consistent with previous reports [9, 14]. This finding supports the statement that women’s cognitive control to follow the instructions varies with the level of estradiol [9]. Thus, Hjelmervik et al. [9] concluded that cognitive control can be a confounding factor of lateralization studies. In addition, the current participants exhibited more errors in the forced left condition. Apparently, the cognitive control involved in the task was the most difficult task for them. However, they did improve in the task as their error rate improved across the sessions, indicating that they acclimated to the task over the sessions. This conclusion supports Hiscock and Kinsbourne’s [1] statement that the cognitive control to attend to the left ear should not be considered a stable phenomenon.

A limitation of the current study is the fact that hormone levels of the subjects were not directly measured. Future studies should include collection of blood samples and conduction of hormone assays so that peak ovarian hormone conditions can be directly measured instead of estimated based on cycle day. Using the day of the menstrual cycle results in imprecise determination of the hormonal levels, the assumed peak hormone days may have been inaccurate. Also, the selection of pregnancy risk rather than phases of the menstrual cycle limited the ability to directly compare the results of the present study to some of the previous ones. Further research could also be conducted to investigate the effects of the variations in ovarian hormones across the menstrual cycle on auditory sensitivity as measured by otoacoustic emission recording, pure tone audiometry, and auditory brainstem responses.

In conclusion, data from this study indicated no dichotic listening response differences between women taking oral contraceptives and those who did not. These women exhibited laterality quotients consistent with data from previous studies. They exhibited lower REA during the menstrual phase when the level of estrogen began to rise. This lower REA was associated with an increased error rate. In addition, these women exhibited a lower REA in the forced left condition, when cognitive control was being assessed. Along with the lower REA was an increased error rate. However, the women adapted to the task and exhibited lower error rates in this condition across sessions.

## Supporting information

S1 File Raw data from listener responses. The data for the two participant groups across the three presentation conditions and their laterality.
(XLSX)
